# Fibroid Degeneration During Pregnancy Presenting as Appendicitis

**DOI:** 10.7759/cureus.57660

**Published:** 2024-04-05

**Authors:** Jamie Green, Alejandro Biglione

**Affiliations:** 1 College of Medicine, Nova Southeastern University Dr. Kiran C. Patel College of Osteopathic Medicine, Fort Lauderdale, USA; 2 Internal Medicine, Wellington Regional Medical Center, Wellington, USA

**Keywords:** myxoid degeneration, acute abdominal pain, pregnancy, degeneration, fibroid

## Abstract

Uterine fibroid degeneration is a rare cause of abdominal pain during pregnancy. It can cause complications during pregnancy, including placental abruption, fetal growth restriction, and preterm delivery. Myxoid degeneration is an unusual form of fibroid degeneration. We present a case of a 38-year-old female, G1P0, who presented at 13 weeks gestation to the emergency department at the request of her obstetrician due to abdominal pain with concern about appendicitis. A diagnosis of myxoid degeneration was made. The patient was treated with analgesics and discharged to continue her management in the outpatient setting.

## Introduction

Uterine fibroids (leiomyomata) are benign smooth muscle tumors of the uterus. They are the most common pelvic neoplasm in females, with 20-25% of women developing uterine fibroids during their reproductive years [1,2]. They are the leading indication for hysterectomy in the United States. The prevalence of fibroids increases with maternal age [1]. Symptoms can include heavy or prolonged menstrual bleeding and bulk-related symptoms [3]. 

Uterine fibroids can cause subfertility. The size and location of fibroids determine their impact on fertility [4]. The prevalence of fibroids in pregnant women aged 35-42 is 32% [3]. Patients with multiple fibroids or fibroids greater than 5 cm are at increased risk for complications during pregnancy, including placental abruption, fetal growth restriction, and preterm delivery [5,6]. About 10-30% of women with fibroids develop related complications during pregnancy. Fibroid degeneration is one of the possible complications [5]. This case report is about a complicated fibroid during pregnancy presenting with right-lower-quadrant pain mimicking appendicitis. 

## Case presentation

We present a case of a 38-year-old female G1P0 who was 13 weeks pregnant. She presented to the Emergency Department (ED) at the request of her obstetrician for evaluation of abdominal pain with concerns for acute appendicitis. The patient described her pain as dull in character, localized to the right lower quadrant, with an onset five days prior to presentation. The pain was of severe intensity and without any clear precipitating events. The pain was insignificantly decreased by oral analgesics. The patient also reported intermittent mild vaginal bleeding, which was described as brown and without clots. She denied nausea, vomiting, or fever. On her physical examination, she was afebrile, and her vital signs were stable. Her abdominal examination was remarkable for a pregnant uterus, with tenderness to palpation in the right lower quadrant without rebound tenderness, and normal bowel sounds. Her initial laboratory studies showed a normal complete blood count, without leukocytosis and normal blood chemistries.

**Figure 1:**
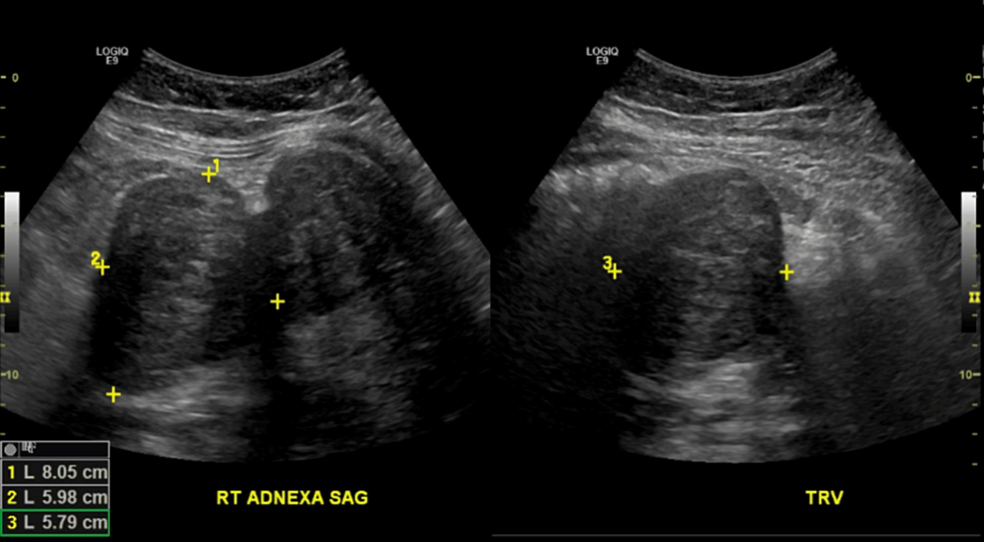
Transabdominal ultrasound

**Figure 2:**
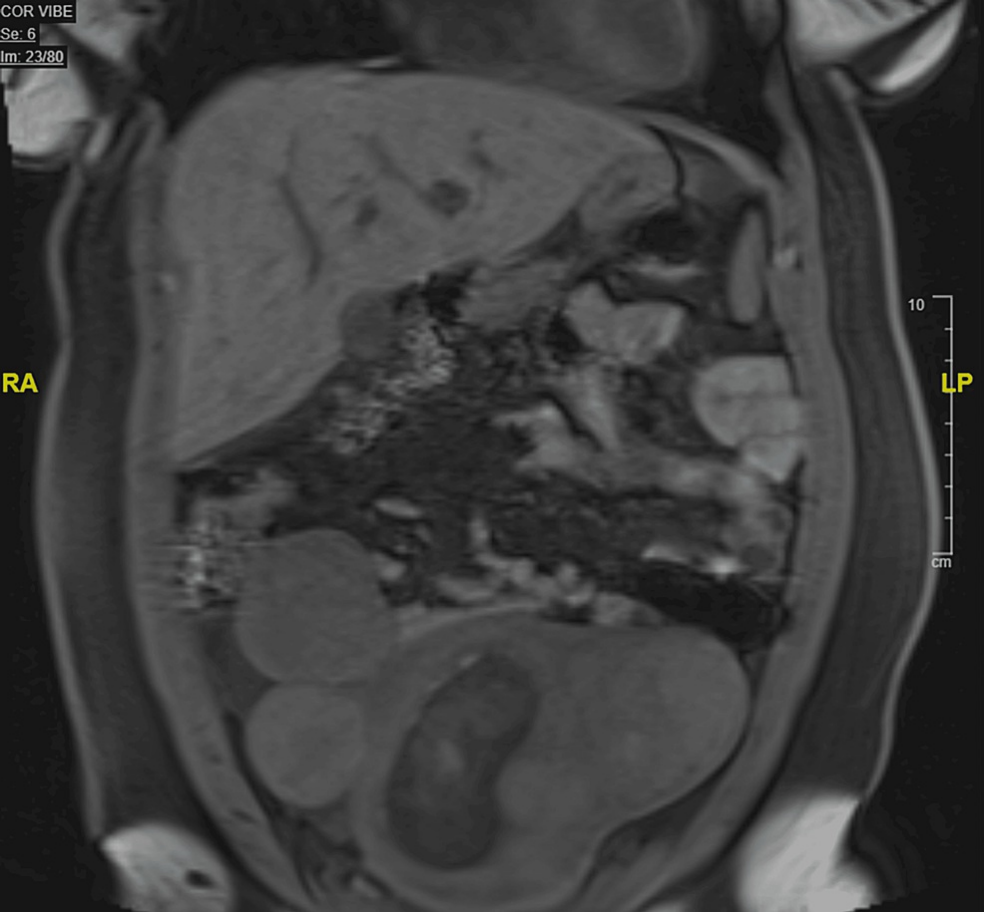
Abdominal/pelvic magnetic resonance imaging without contrast, coronal T1 (volumetric interpolated breath-hold examination) sequence

**Figure 3:**
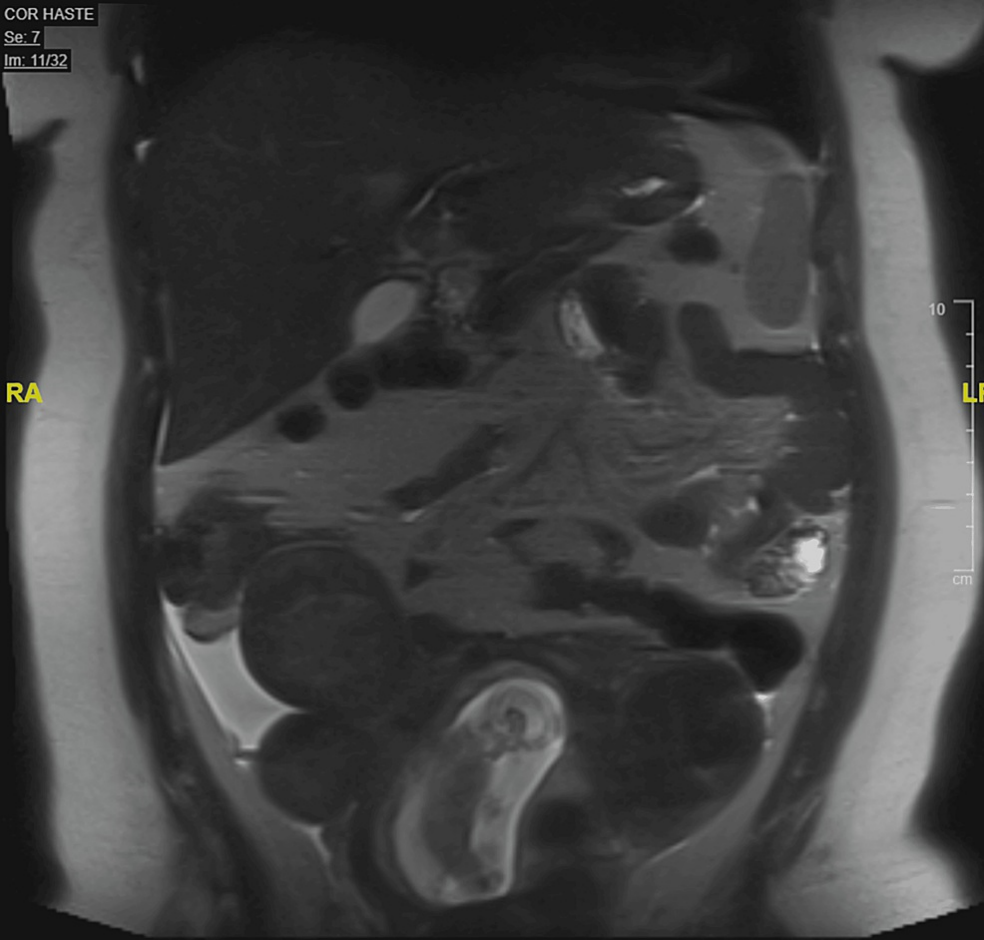
Abdominal/pelvic magnetic resonance imaging without contrast, coronal T2 (half-Fourier acquisition single-shot turbo spin echo) sequence

**Figure 4:**
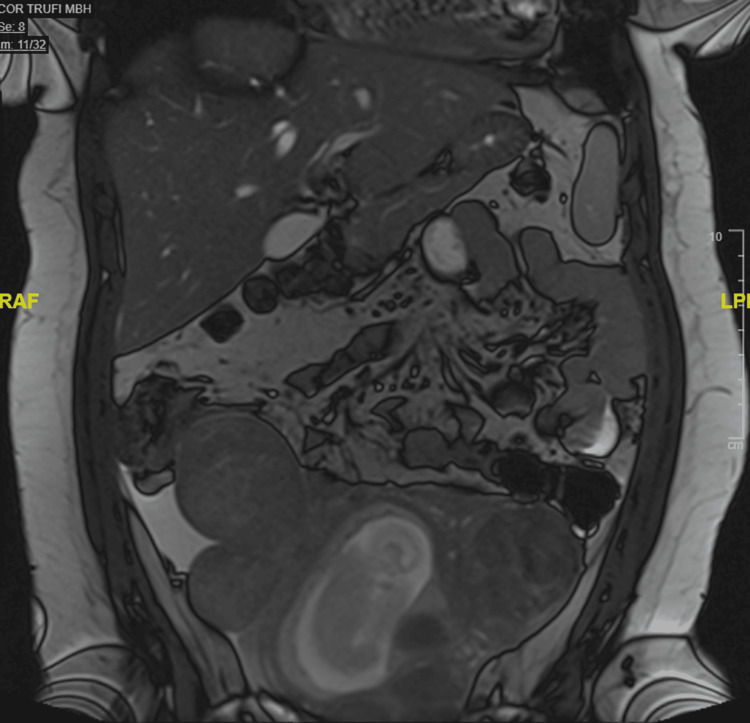
Abdominal/pelvic magnetic resonance imaging without contrast, coronal T2 (true fast imaging with steady-state free precession) sequence

In the ED, a transabdominal ultrasound was performed and showed multiple pedunculated intramural and submucosal fibroids (Figure 1). She also had magnetic resonance imaging (MRI) of the abdomen and pelvis without contrast that revealed the presence of multiple pedunculated uterine fibroids and smaller fibroids in submucosal and intramural locations (Figures 2-4). The MRI also revealed a significant amount of free fluid in the peritoneum, predominantly in the right lower quadrant.

The patient was admitted to the hospital and managed conservatively with intravenous fluids and analgesics as needed. The patient did not display any worsening signs or symptoms of acute abdomen and was discharged the next day on oral analgesics to continue her management in the outpatient setting.

## Discussion

The differential diagnoses of acute abdominal pain during pregnancy are extensive. Degenerated fibroids are an uncommon cause of acute abdominal pain in a pregnant patient. Myxoid degeneration is one of the rarer types of degeneration. In this type of degeneration, the fibroids typically have a myxoid matrix and are filled with a hypocellular, gelatinous material [7]. Diagnosing myxoid degeneration clinically presents challenges due to its infrequent occurrence and the limited distinct signs and symptoms.

Degeneration of uterine fibroids can present similarly to appendicitis, given the sudden and severe abdominal pain in both clinical scenarios. Acute appendicitis is the most common nonobstetric condition requiring surgery during pregnancy [8]. Fever and leukocytosis are poor indicators in pregnancy and should not be used as exclusion criteria, making this diagnosis more difficult in this patient population [9]. In addition, anatomical and physiological changes can hinder the diagnosis, necessitating a high level of suspicion. An unruptured appendix is associated with a fetal loss rate of 1.5-9%, which increases to up to 36% with perforation [8].

In previous cases in which degenerated fibroids clinically imitated appendicitis, the diagnosis was made with transabdominal or transvaginal ultrasound [8]. On ultrasound, degenerated fibroids present as heterogeneous, mostly isoechoic round masses. MRI can be used to confirm the diagnosis as this modality is superior in assessing the size, number, and location of multiple fibroids [10]. On MRI, T1 and T2 sequences display mostly heterogeneous, low-signal intensity.

Myxoid degeneration is usually self-limiting and treated with analgesics. Distinguishing this diagnosis promptly potentially avoids unnecessary surgery.

## Conclusions

Uterine fibroids are common among women of reproductive age. During pregnancy, there is a risk of complications, including myxoid degeneration. This condition should be included in the differential diagnosis of a pregnant woman presenting with acute abdominal pain. Diagnosis is made with ultrasound and/or MRI, and the treatment is symptomatic with analgesics. The prognosis is usually good and without any major threats to the patient or fetus.
